# Interaction of the Macrophage and Primitive Erythroid Lineages in the Mammalian Embryo

**DOI:** 10.3389/fimmu.2016.00669

**Published:** 2017-01-09

**Authors:** James Palis

**Affiliations:** ^1^Department of Pediatrics, Center for Pediatric Biomedical Research, University of Rochester Medical Center, Rochester, NY, USA

**Keywords:** primitive erythropoiesis, macrophage, erythroblastic island, embryo, pyrenocyte

## Abstract

Two distinct forms of erythropoiesis, primitive and definitive, are found in mammals. Definitive erythroid precursors in the bone marrow mature in the physical context of macrophage cells in “erythroblastic islands.” In the murine embryo, overlapping waves of primitive hematopoietic progenitors and definitive erythro-myeloid progenitors, each containing macrophage potential, arise in the yolk sac prior to the emergence of hematopoietic stem cells. Primitive erythroblasts mature in the bloodstream as a semi-synchronous cohort while macrophage cells derived from the yolk sac seed the fetal liver. Late-stage primitive erythroblasts associate with macrophage cells in erythroblastic islands in the fetal liver, indicating that primitive erythroblasts can interact with macrophage cells extravascularly. Like definitive erythroblasts, primitive erythroblasts physically associate with macrophages through α4 integrin–vascular adhesion molecule 1-mediated interactions and α4 integrin is redistributed onto the plasma membrane of primitive pyrenocytes. Both *in vitro* and *in vivo* studies indicate that fetal liver macrophage cells engulf primitive pyrenocytes. Taken together, these studies indicate that several aspects of the interplay between macrophage cells and maturing erythroid precursor cells are conserved during the ontogeny of mammalian organisms.

## Introduction

Two distinct forms of erythropoiesis, primitive and definitive, occur sequentially during ontogeny. Both forms of erythropoiesis consist of lineage-specific progenitors that give rise to maturing erythroblasts that enucleate to form reticulocytes, which mature into red blood cells, and into pyrenocytes, which are rapidly engulfed by macrophage cells. Definitive erythroblasts mature extravascularly in the fetal liver and postnatal bone marrow in physical contact with macrophage cells in erythroblastic islands. The function of these central macrophage cells is poorly understood and currently under active investigation. In contrast to definitive erythropoiesis, primitive erythroid progenitors emerge within the forming vasculature of the yolk sac and give rise to primitive erythroblasts that mature within the fetal bloodstream. Macrophage potential first emerges in the yolk sac and macrophage progenitors seed the liver prior to the emergence of hematopoietic stem cell (HSC). It is now recognized that late-stage primitive erythroblasts interact physically with macrophage cells within erythroblastic islands of the fetal liver. The emergence and interaction of the primitive erythroid and macrophage lineages is the subject of this review.

## Ontogeny of Erythropoiesis

### Primitive Erythropoiesis

Studies conducted more than a century ago identified the emergence of pools of large nucleated erythroid cells in yolk sac blood islands as the first cellular evidence of hematopoiesis (1). These pools of “primitive” erythroid cells rapidly become enveloped by endothelial cells that give rise to a plexus of blood vessels in the yolk sac (2). Maturing primitive erythroblasts arise from a transient population of primitive erythroid progenitors (EryP-CFC) that are first detected at embryonic day 7.5 (E7.25) in the yolk sac of the mouse embryo (3, 4). Murine EryP-CFC are defined by their capacity to generate colonies that contain hundreds of primitive erythroblasts within 5 days of *in vitro* culture in semisolid media. The number of EryP-CFC increase transiently in the yolk sac between E7.25 and E8.5 and subsequently generate a cohort of maturing primitive erythroblasts that begin to circulate into the embryo proper with the onset of the cardiac contractions (5, 6).

**Figure 1:**
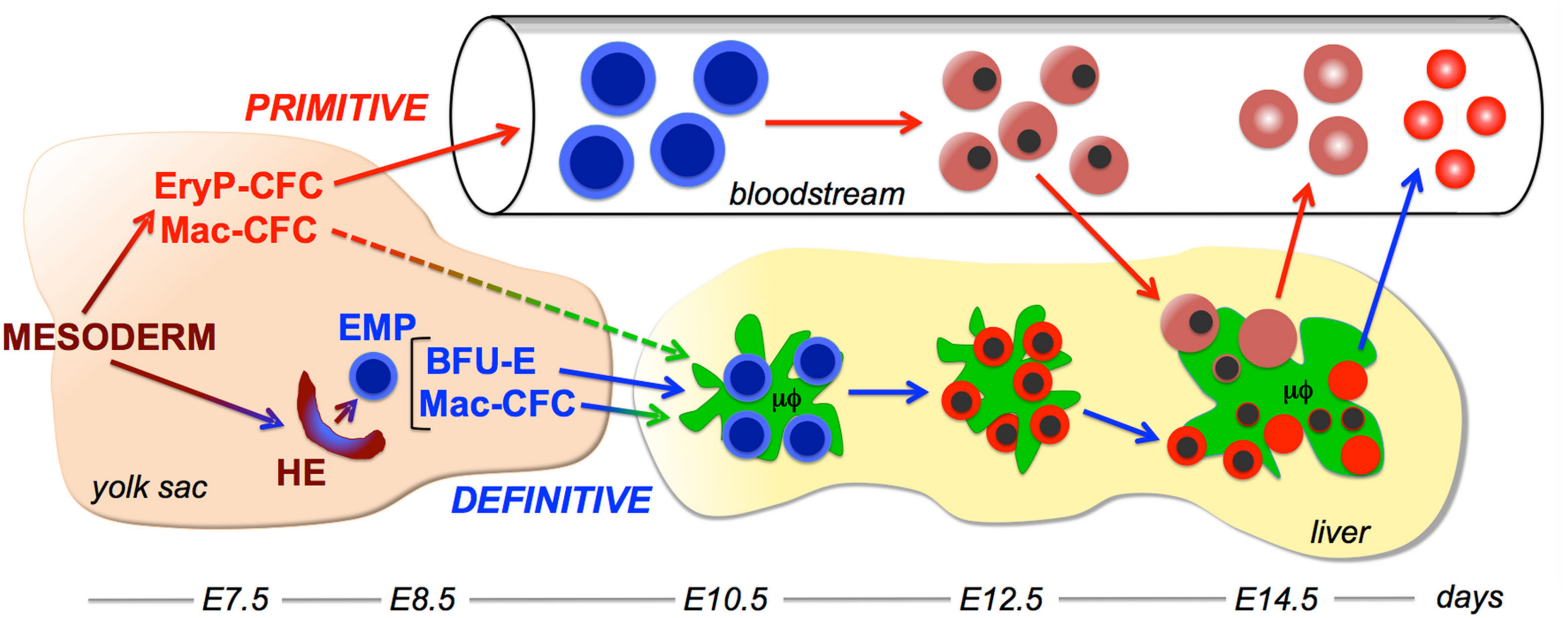
**Working model of interactions between the primitive erythroid and macrophage lineages**. Two overlapping waves of hematopoietic progenitors, primitive hematopoietic progenitors and definitive erythro-myeloid progenitors (EMP) emerge in the yolk sac from extraembryonic mesoderm cells that egress through the posterior primitive streak. Primitive erythroid progenitors (EryP-CFC) give rise to a cohort of primitive erythroblasts that progressively mature in the bloodstream. EMP contain definitive erythroid progenitor [burst-forming units erythroid (BFU-E)] and macrophage progenitor (Mac-CFC) potential. EMP emerge from hemogenic endothelium (HE) in the yolk sac and contain both definitive erythroid (BFU-E) and macrophage (Mac-CFC) potential. EMP, BFU-E, and Mac-CFC seed the liver where they give rise to the first maturing definitive erythroblasts and macrophage cells. Macrophage cells in the liver support definitive erythropoiesis by forming erythroblastic islands. Late-stage primitive erythroblasts enter the fetal liver and physically interact with macrophage cells. Following their enucleation, both primitive and definitive reticulocytes enter the bloodstream, while the resulting pyrenocytes are engulfed by macrophage cells. Embryonic days (E) of development in the murine embryo are provided at the bottom of the figure.

Primitive erythroblasts in the mouse embryo mature as a semi-synchronous cohort within the bloodstream. They expand in numbers while undergoing progressive changes in morphology and gene expression, consistent with a transition from immature (proerythroblasts) at E9.5 to late-stage (orthochromatic) erythroblasts at E12.5 (7) (Figure 1). These morphologic changes include a decrease in cell size, nuclear condensation, and the loss of cytoplasmic basophilia due to the accumulation of hemoglobin and the loss of RNA content. In addition, primitive erythroblasts lose nuclear histone proteins and also lose the intermediate filament vimentin, which permits movement of the nucleus within the cell to facilitate enucleation (8, 9). In the murine embryo, primitive erythroblasts rapidly upregulate and dynamically express primarily embryonic (βH1 and εy) globin genes, which distinguishes them from definitive erythroblasts, which exclusively express adult (β1 and β2) globin genes (10). Primitive erythroid cells are considerably larger than their definitive counterparts in the fetus and ultimately contain fourfold to fivefold more hemoglobin (11).

These maturational changes culminate in the generation of late-stage primitive erythroblasts, which go on to enucleate between E12.5 and E16.5 (12, 13). The expression of the transferrin receptor and α4 integrin are lower in primitive reticulocytes compared to late-stage erythroblasts, consistent with the partition of these molecules to nascent pyrenocytes prior to enucleation (13). During this time period (E12.5–E16.5), primitive reticulocytes increase the physical association of their cytoskeletal network with the outer membrane bilayer, thus gaining increased cellular deformability (14). Like definitive reticulocytes, primitive reticulocytes significantly decrease their total cell volume and surface area as they mature (14). Fully mature primitive erythrocytes continue to circulate throughout the remainder of murine embryogenesis and even persist for several days after birth (12, 13).

Both megakaryocyte and macrophage (Mac-CFC) progenitors emerge in the yolk sac concomitantly with EryP-CFC (4, 15, 16). These findings, supported by studies of *in vitro* differentiated human embryonic stem cells (17), support the notion that primitive hematopoiesis is restricted to the primitive erythroid, macrophage, and megakaryocyte lineages. The first blood cells in the yolk sac are ultimately derived from mesoderm cells that emerge from the posterior primitive streak during gastrulation. *In vitro* studies of murine embryonic stem cells led to the identification of blast colony-forming cells (Blast-CFC) that contained hematopoietic, endothelial, and smooth muscle potential (18). Blast-CFC were subsequently identified in the primitive streak of E7.5 mouse embryos, suggesting that primitive hematopoiesis arises from hemangioblasts (19). In addition, primitive erythroid potential was found to emerge from cells with endothelial identity (20). However, fate-mapping studies of *in vitro* cultured mouse embryos failed to establish clonal relationships between blood islands and their surrounding vasculature and cell-labeling studies did not mark both blood and endothelial cells within the yolk sac (21, 22). These studies, taken together, suggest that if hemangioblasts or hemogenic angioblasts do exist, they constitute rare and/or extremely transient cell populations during gastrulation.

### Emergence of Definitive Erythropoiesis

Analysis of hematopoietic progenitors in embryonic time and space revealed that the first definitive erythroid progenitors, termed burst-forming units erythroid (BFU-E), also arise in the yolk sac (3, 4). Murine BFU-E are distinguished from EryP-CFC by their ability to generate colonies of several 100 definitive erythroid cells in 7–10 days of *in vitro* culture in semisolid media. BFU-E expand in numbers within the yolk sac of the mouse embryo beginning at E8.25, enter the bloodstream, seed the newly forming liver as early as E10.5, and mature to generate the first circulating definitive erythrocytes, which are first evident at E11.5–E12.5 (23) (Figure 1). Ncx-1-null mouse embryos, which lack a beating heart and therefore a functional circulation, contain normal numbers of BFU-E in the yolk sac but fail to seed the liver with hematopoietic progenitors (24). These findings indicate that yolk sac-derived BFU-E jump-start blood cell production in the fetal liver prior to the seeding of the liver by HSC. The lymphangiogenic growth factor VEGF-C is necessary for the appropriate expression of α4 integrin on erythro-myeloid progenitors (EMP) and for EMP colonization of the fetal liver (25). Interestingly, deletion of VEGF-C after midgestation or in the adult does not cause anemia, indicating a specific role for this cytokine signaling pathway in yolk sac-derived erythropoiesis. Consistent with murine studies, limited investigation of human embryos indicate that BFU-E also first emerge in the yolk sac and colonize to the fetal liver prior to HSC emergence (26, 27).

The emergence of BFU-E in the murine embryo is associated both temporally and spatially with the emergence of megakaryocyte, macrophage (see below), granulocyte, and mast cell lineages (4, 15, 16). A multipotential hematopoietic progenitor cell with definitive erythroid, megakaryocyte, and multiple myeloid lineage components, termed the EMP, has been identified in the E9.5–E10.5 murine yolk sac by the concomitant cell surface expression of kit, CD41, and CD16/32 (28–31). Clonal studies indicate that single EMP can contain both definitive erythroid and myeloid lineage potential (31).

Hematopoietic stem cells emerge in endothelial-associated cell clusters in large arterial vessels through a Runx1-dependent endothelial-to-hematopoietic transition (32, 33). Interestingly, EMP also emerge in cell clusters, which are localized to the yolk sac in kit+Runx1+ cells (34, 35). In addition, like HSC, EMP require Runx1 for their emergence, providing further evidence that EMP emerge *via* an endothelial-to-hematopoietic transition (35, 36). Recent studies indicate that EMP and some HSC, but not primitive erythroid cells, emerge from LYVE-1+ endothelium (37). These results point to the distinct developmental origins of primitive- and EMP-derived hematopoiesis from posterior mesoderm (Figure 1).

## Ontogeny of the Macrophage Lineage(s)

The first myeloid cell potential in the murine embryo consists specifically of macrophage progenitors (38). Mac-CFC first emerge and expand the number within the yolk sac concomitant with primitive erythroid progenitors (4) (Figure 1). These initial Mac-CFC give rise directly to macrophage cells, rather than going through a monocyte intermediate (39). Mac-CFC continue to expand in numbers in the yolk sac concomitant with definitive erythroid progenitors, and like BFU-E, are subsequently found in the bloodstream and fetal liver (4, 38). These kinetics indicate that the macrophage lineage, as has also been found for the megakaryocyte lineage, is a component both of the first (primitive) and of the second (EMP) waves of hematopoietic progenitors that emerge in the yolk sac (Figure 1).

The first immature embryonic macrophages are detected in the murine yolk sac at E9.0 (40–42). Soon thereafter, macrophage cells become widely distributed throughout the embryo, with the highest concentration localized within the fetal liver (Figure 1). It is now recognized that these yolk sac-derived macrophage cells give rise to multiple long-lived tissue-resident macrophage populations, including microglia in the brain, Langerhans cells in the skin, and Kupffer cells in the liver (43–45). Tissue-resident macrophages express organ-specific genes that are regulated by distinct enhancer profiles (46, 47). The tissue-specific gene expression programs of tissue-resident macrophage cells become specified soon after macrophage cells seed organs during embryogenesis (48). However, the developmental origin and specific gene expression program of macrophage cells associated with erythroblastic islands remain poorly understood. Tissue-resident macrophage cells also use a self-renewal program identified in embryonic stem cells to maintain their long-term presence within tissues (49).

## Interactions of Macrophage Cells and Definitive Erythroblasts

Pioneering studies by Bessis and Mohandas indicated that all erythroblasts in the bone marrow mature within “erythroblastic islands” consisting of central macrophages that extend cytoplasmic projections to a ring of surrounding erythroblasts (50, 51). Erythroblastic islands had not been previously recognized because they are physically disrupted when smears are prepared from the marrow. However, after gentle physical or enzymatic disruption, erythroblast islands in the bone marrow or the fetal liver can be identified and enumerated (52, 53). Erythroblastic islands can be reconstituted *in vitro* when erythroblasts are coincubated with freshly isolated macrophage cells (54).

The isolation of intact erythroblastic islands and their physical reconstitution indicate that adhesive interactions occur between erythroid precursors and macrophage cells. Several adhesion molecules have been identified that mediate these interactions. Adhesive interactions occur between α4β1 integrin expressed on erythroblasts and its counter-receptor vascular adhesion molecule 1 (VCAM-1) expressed on macrophage cells. Monoclonal antibodies directed against β1 integrin or VCAM-1 each disrupt erythroblastic island formation *in vitro* (55).

Another adhesive interaction between definitive erythroid cells and macrophage cells is mediated by erythroblast-macrophage protein, which is encoded by Maea. Maea is a 36-kD transmembrane protein expressed both by erythroblasts and macrophage cells (56, 57). Culture of human erythroblasts without macrophage cells or in the presence of macrophage cells and anti-MAEA antibodies resulted in a marked decrease in erythroblast proliferation and enucleation and a marked increase in erythroblast apoptosis (56). MAEA-null mouse fetuses have increased numbers of nucleated erythroid cells in the bloodstream and die of anemia before birth (58). *In vitro* erythroblast island reconstitution assays indicate that MAEA function is mediated by its expression both on erythroid cells and on macrophage cells.

Yet another adhesive interaction is the erythroid-specific isoform of intercellular adhesion molecule 4 (ICAM-4), which interacts with αv integrin on macrophage cells (59), as well as the β1 integrin on leukocytes and the platelet integrin αIIβ3. The addition of synthetic αv peptides blocks this interaction and disrupts erythroblastic island integrity *in vitro* (53). Targeted disruption of ICAM-4 causes a 50% reduction in the number of erythroblastic islands in the marrow; however, steady-state erythropoiesis is not adversely affected in adult ICAM-4-null mice (53).

### Functions of Erythroblastic Islands

Definitive erythroblasts can proliferate, mature, and enucleate *in vitro* in the absence of other cell types. However, this process is inefficient and terminal erythroid maturation can be enhanced by coculture with accessory cells (60, 61). Electron-microscopic studies of erythroblastic islands have suggested that macrophages may “nurse” erythroblasts by supplying them with iron (62). This process was subsequently recognized to be micropinocytosis, a process by which immature erythroblasts accumulate iron through a specific acid ferritin receptor (63). It has also been proposed that central macrophage cells may serve as an important source of cytokines, in particular EPO, that support erythroid maturation (64). Consistent with this hypothesis, the coculture of erythroblasts with macrophage cells prevents erythroblast apoptosis (57).

The *in vitro* coculture of macrophage cells with human and murine definitive erythroblasts results in increased numbers of maturing erythroid cells (56, 61). The chronic depletion of macrophage cells in adult mice led to decreased phagocytosis of senescent red blood cells resulting in a prolonged red cell lifespan and a compensatory decrease in red blood cell output from the bone marrow. However, the response to the induction of acute anemia is blunted in macrophage-depleted mice (65, 66). While controversial, these findings suggest that macrophage cells, or potentially dendritic cells (67), which are also depleted by clodronate, play an important role in the stress response of adult mice (68).

**Figure 2:**
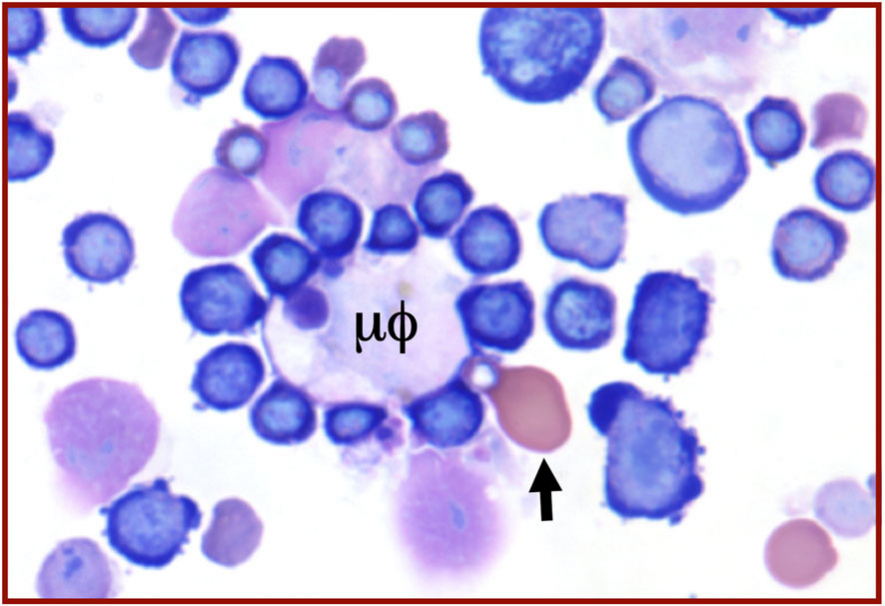
**Wright–Giemsa stain of murine fetal liver cells**. An erythroblastic island with a central macrophage (μϕ) to which are attached many intermediate-stage definitive erythroblasts, well as one late-stage, well-hemoglobinized primitive erythroblast (arrow).

The attachment to numerous erythroblasts to a central macrophage brings erythroblasts into close physical proximity (Figure 2). This proximity facilitates erythroblast–erythroblast interactions, such as the regulation of erythroid cell numbers by Fas–FasL signaling. Expression of FasL on late-stage erythroblasts can transmit a death signal to adjacent immature erythroblasts that express Fas (69). High levels of EPO protect immature erythroblasts from such death signals, leading to increased erythroid cell survival (69, 70).

Finally, macrophage cells play an important role in erythropoiesis by engulfing and digesting pyrenocytes derived from the process of enucleation. This engulfment is mediated by macrophage responses to the display of an “eat me” signal, phosphatidylserine, along with Protein S, on the surface of pyrenocytes and involves the recognition of the latter by the Mer tyrosine kinase receptor on macrophage cells (71, 72). Digestion of the nucleus present in the engulfed pyrenocyte requires the enzyme DNAse II, which is upregulated in fetal liver macrophages by the transcription factor Klf1 (73). While Klf1 was considered to be an exclusively erythroid-specific transcription factor, it is also expressed by hematopoietic progenitors (EMP) in the yolk sac that give rise to the macrophage cells that seed the fetal liver (74). Thus, Klf1 is responsible not only for intrinsically regulating the maturation of primitive and definitive erythroid cells (75, 76), it also regulates the tissue-specific gene expression program in the macrophage cells that interact with maturing erythroblasts. Targeted disruption of DNAse II leads to macrophage cells in the fetal liver that becomes massively engorged with erythroblast nuclei, resulting in the expression of autoimmune-related cytokines, severe fetal anemia, and ultimately perinatal death (77, 78). These findings speak to the importance of pyrenocyte clearance by macrophage cells.

Two genes have been implicated in the regulation of definitive erythroid and macrophage lineages that regulate their interactions within erythroblastic islands. The first is retinoblastoma (Rb), which regulates the G1-to-S-phase transition of the cell cycle. Targeted disruption of Rb results in fetal anemia, though its preferential requirement in definitive erythroid versus macrophage cells remains controversial (54, 79–81). The second is Tropomodulin 3 (Tmod3) that binds tropomyosins and regulates the length and stability of actin filaments. Targeted disruption of Tmod3 induces multiple defects of definitive erythroid maturation in the fetal liver, disrupts formation of erythroblastic islands, and ultimately causes fetal demise between E14.5–E18.5 (82). Interestingly, *in vitro* reconstitution studies indicate that Tmod3 is required both in definitive erythroid cells and in macrophage cells.

The importance of macrophage cells for the terminal maturation of definitive erythroid cells in the fetal liver is evidenced by the progressive fetal anemia and fetal death of Palladin (Palld)-null embryos (83). Targeted loss of this actin cytoskeleton-associated protein results in the disruption of erythroblastic islands in the fetal liver and a marked lack of definitive, but not primitive, erythroid cells in the E13.5–E14.5 mouse embryo. *In vitro* erythroblastic island reconstitution studies indicate that Palladin function is specifically required in macrophage cells.

## Interactions of Primitive Erythroblasts with Macrophages in the Fetal Liver

The liver serves as the site of fetal erythropoiesis, which is colonized by macrophage cells before the appearance of differentiating definitive erythroblasts (84). BFU-E are found as early as E10.5 in the murine fetal liver and the number of cells in the liver expands exponentially between E11.5 and E15.5, reflecting a massive increase in definitive erythropoiesis (85). These maturing definitive erythroblasts are localized near macrophage cells within erythroblastic islands (86). Immunohistochemical studies of E15.5 fetal livers revealed the spatial association not only of definitive erythroblasts, but also of primitive erythroblasts with F4/80-positive macrophage cells (87). Isolation of erythroblastic islands from E14.5 fetal livers also revealed late-stage primitive erythroblasts attached to central macrophage cells (87) (Figure 2). These findings, along with the disproportionate concentration of nucleated primitive erythroblasts in the E15.5 fetal liver compared to the bloodstream, provided evidence that primitive erythroblasts enter the fetal liver and physically interact with macrophage cells. This conjecture was tested by *in vitro* erythroblastic island reconstitution studies. Late-stage primitive erythroblasts collected from the fetal bloodstream were able to reconstitute erythroblastic islands when incubated with fetal liver-derived macrophage cells that had been stripped of their attached definitive erythroblasts (87, 88). Further evidence of primitive erythroblast–macrophage interactions has come from the identification of erythroblastic islands composed of central macrophage cells surrounded by maturing primitive erythroblasts in cultures of *in vitro* differentiated embryonic stem cells (74).

**Figure 3:**
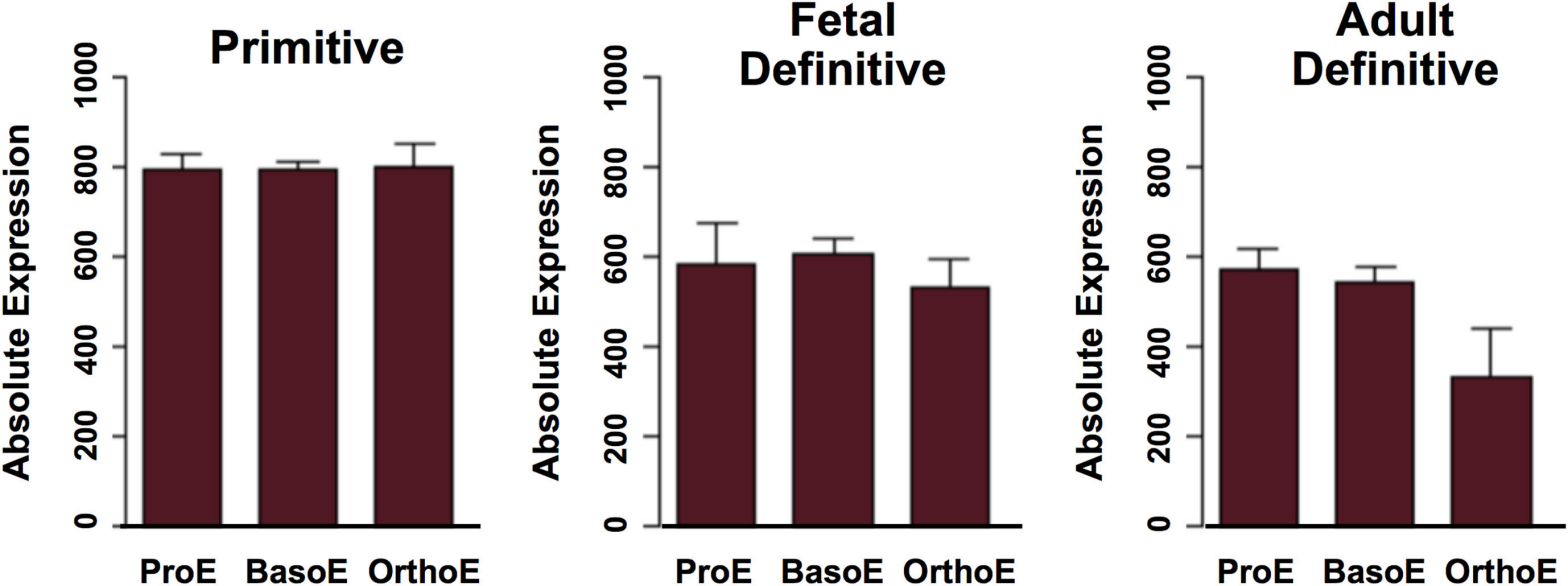
**Expression of Maea gene transcripts in maturing primitive and definitive erythroid cells**. ProE, proerythroblasts; BasoE, basophilic erythroblasts; OrthoE, late-stage polychromatophilic and orthochromatic erythroblasts. Data were derived from http://www.cbil.upenn.edu/ErythronDB (7).

Late-stage primitive erythroblasts in the fetal liver upregulate the surface expression of α4 and α5 integrins (13, 88), suggesting that integrins mediate primitive erythroblast adherence to macrophage cells. This conjecture was tested by the blockade of α4 integrin and of the α4 counter-receptor on macrophage cells, VCAM-1, each of which led to decreased numbers of primitive erythroblasts associated with macrophage cells (87, 88). These findings indicate that α4 integrin–VCAM-1 adhesive interactions function both in primitive and definitive erythropoiesis. While Maea transcripts are expressed at similar levels in primitive and definitive erythroid cells (Figure 3), the potential role of Maea–Maea, or of other adhesive interactions, in primitive erythroblast–macrophage interactions has not, to my knowledge, been reported. The potential roles of Palladin, Rb, and Tmod3 in primitive erythroblast–macrophage interactions remain unknown.

What are the functions of macrophage cell interactions with primitive erythroblasts in the fetal liver? In definitive erythropoiesis, all stages of erythroblast maturation are associated with macrophages in erythroblastic islands (Figure 1). By contrast, EryP-CFC (E7.25–E8.5) and proerythroblast (E9.5) stages of primitive erythropoiesis occur before the fetal liver is formed and primitive erythroblasts isolated from E9.5 mouse embryos do not readily attach to macrophage cells (88). Together with the immunohistochemical and reconstitution studies described above, these findings suggest that only late-stage primitive erythroblasts interact with macrophages cells, unlike definitive erythroblasts that physically interact with macrophage cells throughout their maturation (Figure 1). Therefore, it is unlikely that macrophage cells “nurse” immature primitive erythroblasts with supplies of iron or cytokines.

Unlike definitive erythroblasts, only small numbers of primitive erythroblasts enucleate when cultured *in vitro* (87). The *in vitro* coculture of primitive erythroblasts with macrophage cells enhanced their enucleation (87), although others did not see accumulation of enucleated cells over time (88). The central macrophage cells in coculture experiments took up nuclei derived from primitive erythroblasts and genetically labeled primitive erythroid nuclei were found *in vivo* in fetal liver macrophages (87, 88). These findings indicate that fetal liver macrophage cells normally engulf and digest primitive pyrenocytes. The transient presence of primitive pyrenocytes in the bloodstream of E14.5–E15.5 mouse embryos (87) suggests that their engulfment by macrophage cells in the fetal liver is somewhat inefficient. Alternatively, it is possible that not all primitive erythroid enucleation events occur extravascularly or in the context of erythroblastic islands.

In summary, embryonic macrophage cells and primitive erythroid cells are both derived from hematopoietic progenitors that emerge in the yolk sac. Macrophage cells seed the fetal liver and support the massive expansion of definitive erythropoiesis that occurs there. They also physically bind late-stage primitive erythroblasts that enter the fetal liver relying, in part, on α4 integrin and VCAM-1 interactions. While fetal liver macrophages may enhance enucleation and engulf and digest primitive pyrenocytes, other roles for, and molecules mediating, primitive erythroblast–macrophage interactions remain to be elucidated.

## Author Contributions

JP wrote the article and prepared the figures.

## Conflict of Interest Statement

The author declares that the research was conducted in the absence of any commercial or financial relationships that could be construed as a potential conflict of interest.
